# M1 Muscarinic Receptor Activation Mediates Cell Death in M1-HEK293 Cells

**DOI:** 10.1371/journal.pone.0072011

**Published:** 2013-09-02

**Authors:** E. Scott Graham, Kerhan K. Woo, Miranda Aalderink, Sandie Fry, Jeffrey M. Greenwood, Michelle Glass, Mike Dragunow

**Affiliations:** Department of Pharmacology and Clinical Pharmacology, Centre for Brain Research, School of Medical Sciences, Faculty of Medical and Health Sciences, The University of Auckland, Auckland, New Zealand; St. Jude Children’s Research Hospital, United States of America

## Abstract

HEK293 cells have been used extensively to generate stable cell lines to study G protein-coupled receptors, such as muscarinic acetylcholine receptors (mAChRs). The activation of M1 mAChRs in various cell types *in vitro* has been shown to be protective. To further investigate M1 mAChR-mediated cell survival, we generated stable HEK293 cell-lines expressing the human M1 mAChR. M1 mAChRs were efficiently expressed at the cell surface and efficiently internalised within 1 h by carbachol. Carbachol also induced early signalling cascades similar to previous reports. Thus, ectopically expressed M1 receptors behaved in a similar fashion to the native receptor over short time periods of analysis. However, substantial cell death was observed in HEK293-M1 cells within 24 h after carbachol application. Death was only observed in HEK cells expressing M1 receptors and fully blocked by M1 antagonists. M1 mAChR-stimulation mediated prolonged activation of the MEK-ERK pathway and resulted in prolonged induction of the transcription factor EGR-1 (>24 h). Blockade of ERK signalling with U0126 did not reduce M1 mAChR-mediated cell-death significantly but inhibited the acute induction of EGR-1. We investigated the time-course of cell death using time-lapse microscopy and xCELLigence technology. Both revealed the M1 mAChR cytotoxicity occurs within several hours of M1 activation. The xCELLigence assay also confirmed that the ERK pathway was not involved in cell-death. Interestingly, the MEK blocker did reduce carbachol-mediated cleaved caspase 3 expression in HEK293-M1 cells. The HEK293 cell line is a widely used pharmacological tool for studying G-protein coupled receptors, including mAChRs. Our results highlight the importance of investigating the longer term fate of these cells in short term signalling studies. Identifying how and why activation of the M1 mAChR signals apoptosis in these cells may lead to a better understanding of how mAChRs regulate cell-fate decisions.

## Introduction

The five subtypes (M1–M5) of muscarinic acetylcholine receptors (mAChRs) are widely distributed in the body and are involved in a variety of physiological functions. In the brain, mAChRs mediate the majority of transmission by acetylcholine and are involved in the control of neurological functions such as movement, attention and memory processes [1]. Given the complexity of this system, considerable effort has been focused at understanding the function of each receptor subtype (M1 to M5).

In the central nervous system, the M1 and M3 AChR subtypes have been implicated in the survival of a variety of cell types including neuronal cells [2]. A considerable literature exists for M3 receptors and their role in cell survival [3]–[6] or conversely, in cell death [7]. In contrast, the involvement of M1 AChR in the survival of neuronal cells has not been studied as extensively, but several reports have shown that cholinergic activity mediated through M1 AChRs modulates the survival of retinal ganglion cells [8]–[10].

For more than a decade there has been growing interest in the M1 mAChR as a potential target for drug development in Alzheimer’s disease (for recent review see [11]). The development of M1 selective agonist for AD has been pioneered by these researchers [12], who have focused on developing AD modifying M1 selective drugs with improved brain permeability and pharmacology specific to M1 mAChRs [13], [14]. In a seminal paper published in Neuron, Fisher and colleagues demonstrated an impressive ability of an M1 selective agonist to reverse the amyloid and tau pathology in the triple transgenic AD mouse [15]. Although the exact cellular mechanisms of action are currently unclear, the improved pathophysiological changes were consistent with the M1 agonist reversing the cognitive deficits observed in this model [15].

It has recently been shown that the non-phosphorylated or dephosphorylated tau protein can behave as an M1 and M3 agonist, resulting in prolonged cytoplasmic calcium elevation resulting in neuronal cell death [16]. Liberation of tau proteins may occur as a result of cell death, thus potentially contributing to the exacerbation of neuronal cell loss through muscarinic receptors. The clinical significance of this latter observation has yet to be elucidated but indicates that under certain conditions M1 receptors can mediate cytotoxic effects as well as survival pathways. Such pleiotropic effects have been observed for a number of receptors and are in part dependent on the cell signalling cascades activated and phenotype of activated cells.

HEK293 cells are widely used as a cell-based model for the transfection of various mAChRs including the M3 [17]–[19] and M1 [20], [21] subtype to further study how they respond to agonists and affect cellular functions. Because they have been shown to express low levels of the endogenous M3 mAChR [22] and they faithfully reproduce exogenous levels of mAChRs [23], this model was useful to dissect out the signalling effects of the M1 mAChR associated cell life and death. Given the clinical relevance of M1 AChR in the pathology of various diseases better understanding of M1 mediated cell survival and cell death pathways is clearly warranted. Therefore the aim of this project was to develop a HEK-cell model of M1AChR to investigating the signalling pathways involved in mediating neuroprotection of M1 agonists.

## Materials and Methods

### 2.1 Materials

HEK293 cells (CRL-1573) were purchased from ATCC. Cell culture media components were purchased from Gibco (Invitrogen) and cell culture plastic ware were purchased from Nunc. The M1 mAChR (3x-hemagglutinin (HA.11) tagged at the N-terminus) in vector pcDNA3.1+ (clone MAR010TN00) was purchased from the Missouri S&T cDNA Resource Centre and were sequence verified on arrival. The empty vector was purchased from Invitrogen. Carbachol was purchased from Sigma, 5-bromo-2-deoxyuridine (BrdU) from Roche, 1,4-diamino-2,3-dicyano-1,4-bis[2-aminophenylthio]butadiene (U0126) from Cell Signaling Technology, MT7 from the Peptide Institute and HU210 from Tocris.

The sources of primary antibodies and the species they were derived from for the following proteins are as follows – EGR-1 (rabbit, sc-189) from Santa Cruz Biotechnology, M1 mAChR (rabbit, M9808) from Sigma, HA.11 (mouse, MMS-101P) from Covance, ERK1/2 (rabbit, 9102), phospho-p44/42 ERK1/2 (rabbit, 9101) and cleaved caspase-3 (rabbit, 9661L) from Cell Signaling Technologies, pCREB (rabbit, 06-519) from Upstate Biotechnology, β-Actin (mouse, ab6276) from Abcam, and BrdU (mouse, 11 170 376 001) from Roche.

Peroxidase-conjugated anti-rabbit and anti-mouse (B7389 and B7264), ExtrAvidin horse radish peroxidase (E-2886), chromogen 3,3′-diaminobenzidine (DAB) and the Hoechst nuclear stain (33258) were purchased from Sigma. The Alexa Fluor 488 anti-mouse antibody (A-11029) was purchased from Invitrogen. The Taq polymerase was purchased from Roche and Trizol and all other components for PCR analysis on total RNA were purchased from Invitrogen. All other chemicals were purchased from Sigma or BDH.

### 2.2 Cell Culture

HEK293 cells were stably transfected with the M1 mAChR or the vector alone. These cells are herein referred to as HEK293-M1 and HEK293-Vec respectively, and were cultured using standard techniques. Briefly, transfection involved plating HEK293 cells in a 24 well plate, and transfecting with 800 ng of DNA that was linearised with the Sca*I* enzyme, using Lipofectamine 2000 (Invitrogen) according to the manufacturer’s instructions, and cultured overnight at 37°C in a humidified atmosphere (5% CO_2_/95% air). The next day the cells were trypsinised with 0.05% trypsin and EDTA, washed with media and seeded into a T25 flask in supplemented media (DMEM medium supplemented with 10% fetal bovine serum, 100 U/ml penicillin, 100 µg/ml streptomycin, 2 mM L-glutamine and 200 µg/ml G418), and maintained with regular media changes for 2–3 weeks before they were considered to be stable cell lines. HEK293 cells that were stably transfected with the CB1 cannabinoid receptor (HEK293-CB1) have been previously characterised and described (Grimsey et al, 2008). These transfected cells were grown to confluency then harvested with 0.05% trypsin and EDTA, washed with media and plated out, 18–24 h prior to drug addition, in 96-well plates coated with poly-L-lysine at 1.5×10^4^ cells/well for immunocytochemistry and microscopy. Drugs were dissolved as follows – carbachol in water, U0126 and MT7 were dissolved in DMSO and diluted in supplemented media so that the final concentration of DMSO added to the cells was only 0.1%, BrdU was diluted in supplemented media and HU210 was diluted in ethanol.

### 2.3 RNA Extractions and RT-PCR of Transfected M1 mAChRs

HEK293-Vec and HEK293-M1 cells were harvested using versene, pelleted and snap-frozen on dry-ice. Total RNA was extracted using Trizol following the manufacturer’s instructions (Invitrogen). Then 1 µg of total RNA was DNase I (Invitrogen) treated to remove contaminant genomic DNA. RNA was then reverse-transcribed (RT) into cDNA using Superscript II according to the manufacturer’s instructions (Invitrogen). Control reactions were conducted where Superscript II was omitted (-RT control). PCR primers were as follows: HA-M1 mAChR forward, 5′-GCTTACCCATACGATGTTCCA-3′ (located in HA epitope region of M1) and M1 mAChR reverse 5′-GCTCCTCTCCCTCTGAGGAT-3′ to amplify a 916-bp amplicon; GAPDH forward 5-TGGAAGTCCTCCAAAAGCCCAG-3′, GAPDH reverse 5′-TGTCCATAGACACAGCCCTTCAG -3′ to amplify a 430-bp amplicon. The PCR cycling parameters for M1 mAChR were initial denaturation at 95°C for 2 min, 30 cycles of 95°C for 30 s, 66°C for 30 s, and 72°C for 60 s, with final extension at 72°C for 7 min. The same cycling parameters were used for GAPDH PCR except the annealing Tm was 62°C. These PCR annealing conditions were optimised using a gradient Corbett PCR machine (data not shown). PCR amplicons were separated on a 1% agarose gel and the amplicons were visualised using ethidium bromide using standard procedures.

### 2.4 Immunocytochemistry

Standard immunocytochemical techniques were used in this study, cells were usually permeabilsed with 0.2% triton prior to addition of primary antibodies. In additional experiments, live-labelling of cell surface receptors [24] was conducted and this is clearly defined in the respective figure legend. For intracellular staining of pERK, pCREB, EGR-1 and M1 receptor cells were fixed in 4% paraformaldehyde (PFA) for 20 minutes, rinsed in PBS and then permeabilised with PBS containing 0.2% triton X-100 (PBS-T). Then primary antibodies were diluted in immunobuffer (PBS, 0.2% Triton X-100, 1% goat serum and 0.4 mg/ml merthiolate), and were incubated with the fixed cells overnight at 4°C with gentle agitation. The antibodies were added at the following dilutions: cleaved caspase-3 at 1∶500, M1 mAChR at 1∶200, HA.11 at 1∶15,000, pERK1/2 at 1∶250, and EGR-1 at 1∶2,000. For pERK staining, cells were also treated with 90% cold methanol at −20°C for 10 minutes prior to addition of primary antibodies. This step is essential for good pERK staining. The antibody for BrdU was diluted 1∶500 and in this case, Triton X-100 was withheld from the immunobuffer and PBS washes until secondary antibodies were added. After incubation with the primary antibody, cells were washed with PBS-T and incubated with either goat-derived anti-mouse or anti-rabbit biotin conjugated secondary antibodies diluted 1∶500 in immunobuffer overnight at 4°C with gentle agitation. Cells were washed with PBS-T and incubated with ExtrAvidin horse radish peroxidase, diluted 1∶500 in immunobuffer for 2 h at room temperature with gentle agitation. Cells were washed again with PBS-T and incubated with the DAB stain (0.5 mg/ml DAB, 0.1 M phosphate buffer and 0.01% H_2_0_2_) for 10 min. For fluorescent labelling, after incubation with the primary antibodies, cells were incubated with goat-derived anti-mouse or anti-rabbit Alexa488 secondary antibodies (Invitrogen) diluted 1∶500 in immunobuffer overnight at 4°C with gentle agitation. Cells were then washed with PBS-T and visualised. Nuclei were stained with Hoechst (Sigma) diluted to 20 µM in a buffer containing 10 mM Tris pH 7.4, 200 mM NaCl and 1 mM EDTA, and incubated for 30 min at room temperature with gentle agitation then washed with PBS-T.

To determine the time-course of cleaved caspase 3 expression in HEK293-M1 and control cells we exposed cells to 100 µM carbachol in 96 well plates for varying times (2, 4, 8, 24, and 48 hours). Cells were fixed and immunostained for the detection of cleaved caspase 3 as detailed above. The percent cleaved caspase 3-positive cells were quantified using the Discovery-1 automated microscope and Metamorph image analysis program (see below).

### 2.5 Live-labelling: Receptor Immunocytochemistry

For internalisation studies, a live-labelling method was adopted, allowing access of the HA.11 antibody only to the extracellular epitopes and therefore not intracellular receptors [24]. The HA.11 antibody was added at a dilution of 1∶500 in supplemented media and incubated with the cells for 30 min at 37°C. Cells were washed with media to remove unbound antibodies and fresh growth media was added with drug treatments for 1–2 h, before the cells were fixed in 4% PFA for 20 min and washed with PBS-T with gentle agitation. Cells were incubated with the goat-derived Alexa 488 anti-mouse antibody at a dilution of 1∶500 in immunobuffer overnight at 4°C, then washed with PBS-T and visualised. To determine the time-course of M1 receptor internalisation HEK293-M1 expressing cells were incubated in 96 well plates with carbachol and then cells were fixed either immediately (0 time point) or 5, 10, 30 or 60 minutes after carbachol addition. To detect membrane-bound and intracellular receptors cells were immunolabelled with the HA.11 antibody made up in triton-X100 (to permeabilize the cell membrane). Alexa-488 was used to visualize the receptors and plates were then acquired on the Disocvery-1 automated microscope using the green FITC filter (see below).

### 2.6 Microscopy

Brightfield and fluorescence immunocytochemistry images were acquired using a Leica DMIRB inverted fluorescence microscope. Images were captured using a Leica DC100 camera and imported as TIFF images into Photoshop 7.0 (Adobe) using the DC350F Twain module (Leica) for the DC100 camera.

### 2.7 Discovery-1 High-throughput Quantification and Statistical Analyses

Quantification of staining was determined by capturing images on a Discovery-1 automated fluorescence microscope and analysed using Metamorph image analysis software (Molecular Devices) [25]. Brightfield images were acquired with an exposure time of 3 ms whilst fluorescent images were acquired with an exposure time between 800 to 1200 ms with a peak excitation of 470 nm and peak emission of 535 nm. For all images, 4 sites per well (a 2×2 rectangle) were acquired at 100× magnification to represent the entire well. The images were then processed with Metamorph software using Find Spots, a cell counting assay, which defined limits for cell size and then calculated the number of cells per image. The parameters used for the Find Spots assay are as follows – Hoechst: spot size 17, bright spot intensity 20; cleaved caspase-3: spot size 35, dark spot intensity 80; BrdU: spot size 25, dark spot intensity 100. The results (the number of positively stained cells per image) were then logged in a Microsoft Excel spreadsheet. Cell counts for cleaved caspase-3 and BrdU were taken as a percentage of the total number of cells in the image, which were counted using Hoechst staining.

To quantify the intensity of fluorescent staining with the HA.11 antibody, images were thresholded to only include grey values higher than 263, and analysed using Region Statistics to find the average grey value of the staining. Statistical analysis of quantified data was conducted using Graphpad Prism 4.02 software and plotted as the mean +/− SEM. All statistical analyses were by one-way ANOVA followed by Tukey’s multiple comparison test.

To quantify M1 receptor internalisation we used the *Granularity* module in the Metamorph image analysis program. This module measures granules which are induced when the receptor internalises but does not measure membrane-bound receptors. Using appropriately adjusted parameters to detect granules based on their size and fluorescence intensity the program was run on the internalization image data sets. Results were automatically logged to Excel spreadsheets.

To determine the percentage cleaved caspase 3-positive cells undergoing apoptosis after carbachol in M1- and cDNA-HEK expressing cells, images from plates were acquired using the Discovery-1 automated microscope and quantified using the *Cell Scoring* module in the Metamorph image analysis program. This module counts total cell number (from Hoechst imaged cells) and cleaved caspase 3 positive cells and then determines automatically percent of cells expressing cleaved caspase 3. Results were automatically logged to Excel spreadsheets.

### 2.8 Analysis of M1 Mediated Cell Death using xCELLigence Biosensor

The xCELLigence Biosensor technology was used to profile the response of the M1-HEK cells to carbachol and ascertain the temporal profile of death that occurred. Following trypsinisation cells were seeded into E16 xCELLigence plates (Roche and ACEA) and allowed to grow for 24–30 hours before being treated with various drug cocktails. The growth and survival of the cells was monitored continuously throughout the experiment. Data are presented as Cell Index. Cell Index is a measure of the level of cellular adhesion to the multi-electrode array, which covers the bottom of the well on to which the cells adhere and grow. The greater the Cell Index values the greater the total adhesion of cells present in the wells. Generally, cell number directly correlates with output Cell Index reading until confluency is achieved. Conversely when Cell Index measurements decrease and remain decreased (rather than a transient dip), this indicates a negative response from the cells, where the total level of cellular adhesion has been reduced. Where a drop in Cell Index is sustained (over many hours) this typically indicates that the reduced adhesion is a consequence of cell death. When cell index reaches 0.0 all cells are dead. Any values above 0.0 indicate that living adherent cells are present.

## Results

### 3.1 Verification of M1 mAChR mRNA Expression and Cell Surface Receptor Localisation

**Figure 1:**
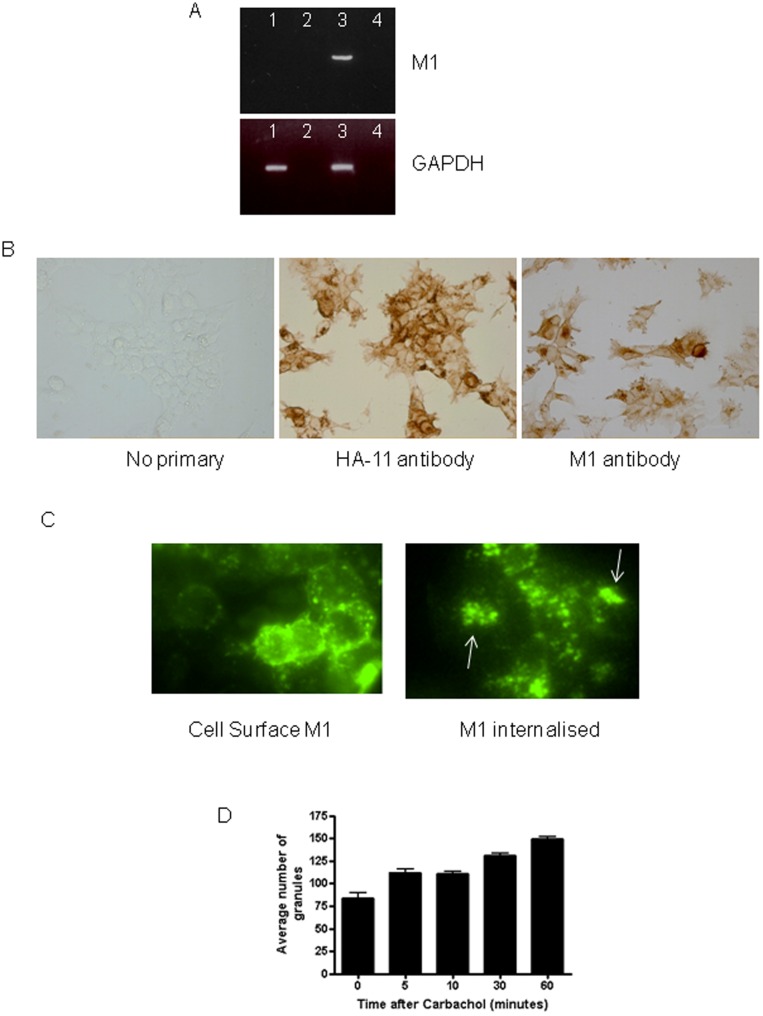
M1 mAChR expression and localisation in transfected HEK293 cells. (A) PCR was conducted to assess M1 expression by vector-transfected (HEK293-Vec) and M1 transfected HEK293 cells (HEK293-M1) using primers for M1 mAChR. The integrity of cDNA samples was confirmed using GAPDH. Samples are as follows; HEK293-Vec cDNA in lane 1, HEK293-Vec -RT/RNA in lane 2, HEK293-M1 cDNA in lane 3, and HEK293-M1–RT/RNA in lane 4. Samples are representative of those used in subsequent functional studies. (B) Immunocytochemical localisation of M1 receptors in HEK293-M1 cells using anti-HA.11 antibody (middle panel) and M1 antibody (right-hand panel). Left panel shows no primary control Image. (C) Analysis of M1 receptor cell-surface expression and internalization by carbachol. Cell surface receptors were live-labeled (see methods) using the HA.11antibody prior to stimulation with water-control or carbachol treatment. The M1 mAChRs were typically localised at the plasma membrane after water treatment, but after 1 h carbachol treatment for the M1 mAChRs were internalised, as shown by the increased punctate cytoplasmic staining (arrows) and reduced staining intensity on the cell surfaces. Scale bar: 50 µm. Data are representative of at least three independent experiments. (D) shows the time-course of M1 receptor internalization after carbachol addition using the granularity assay in Metamorph to measure internalized receptors (as intracellular granules). The graph shows that 5–60 minutes after carbachol addition there is internalization of M1 receptors.

Stable HEK293 cell-lines were prepared expressing the M1 mAChR. In addition, a control cell-line transfected with the empty vector was also prepared (HEK293-Vec). To confirm M1 receptor expression, we conducted PCR and analysed total and cell-surface receptor localisation (Fig. 1). PCR for the M1 mAChR confirmed M1 mAChR mRNA transcripts in HEK293-M1 cells. Control HEK293-Vec cells do not endogenously express M1 mAChR mRNA (Fig. 1A), however they do endogenously express M3 receptors (data not shown). Immunocytochemical analysis of M1 receptors using anti-HA.11 antibodies and M1-specific antibodies revealed localisation of receptors internally and distinct cell-surface M1 receptor expression (Fig. 1B and C). No antibody staining was observed in the HEK293-Vec control cells (Fig. 1B).

The internalisation of G protein-coupled receptors (GPCRs) is integral to their function, serving as a mechanism to down-regulate receptor signalling upon their stimulation with an agonist [26]. To show that the exogenous M1 mAChRs internalise in HEK293 cells following agonist treatment, we performed live-labelling before 100 µM carbachol stimulation, by incubating the cells with the antibody directed to the HA.11 tag. M1 mAChRs were shown to internalise at 1 h after carbachol treatment (Fig. 1C). Internalisation is marked by the reduction in membrane staining and the increased punctate (granular) cytoplasmic staining (Fig.1C). To quantify the internalisation and measure its time-course we undertook time-course studies after carbachol addition to HEK-293-M1 cells (Fig. 1D). M1 receptors internalized by 5-min after carbachol addition for up to 1 hour (the longest time interval tested).

### 3.2 Acute M1 mAChRs Activation Induces ERK1/2 and EGR-1 Induction

**Figure 2:**
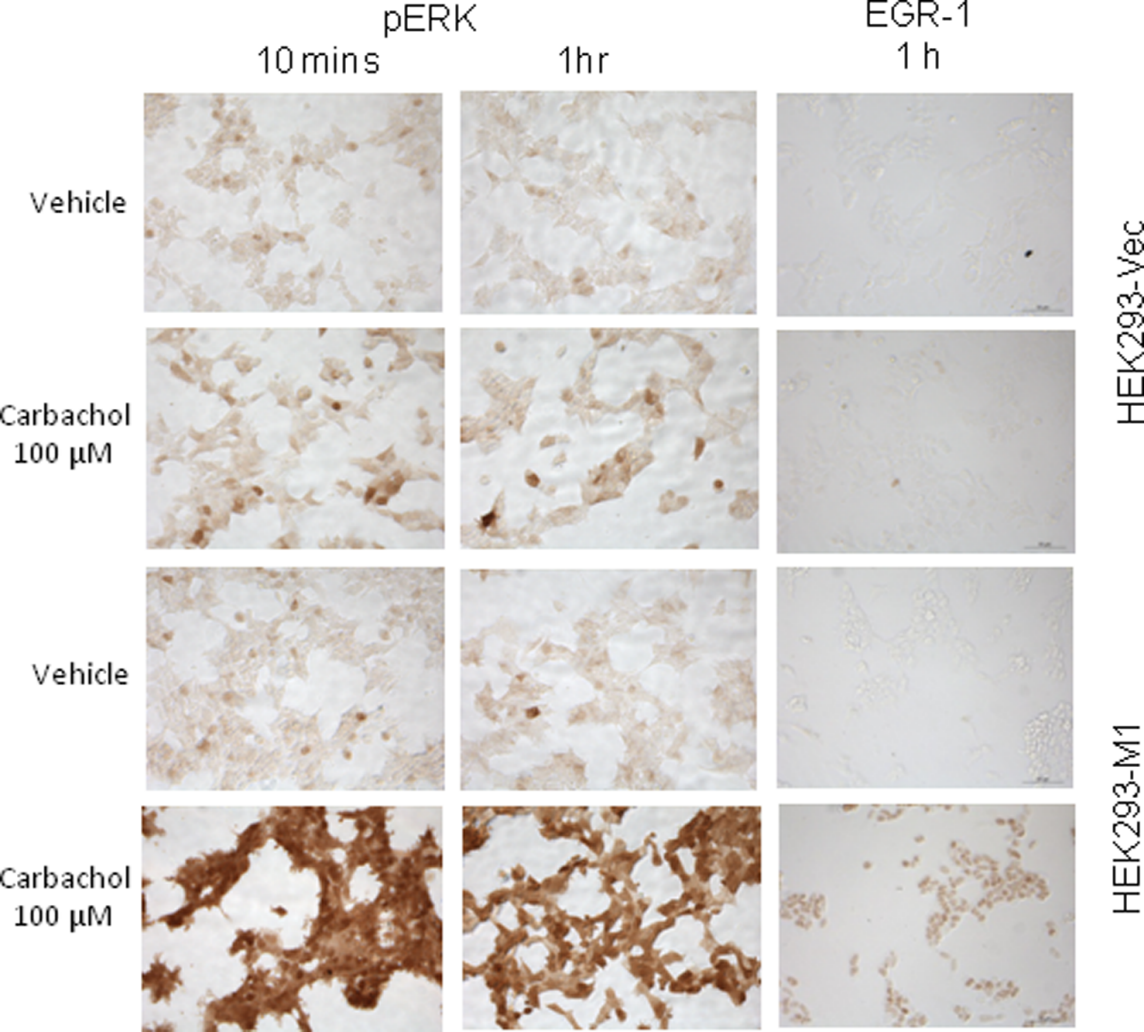
pERK1/2 and EGR-1 induction in transfected HEK293-M1 cells by carbachol. Expression of pERK1/2 and EGR-1 after short term carbachol treatment in HEK293-Vec cells and HEK293-M1 cells. Cells were stimulated for 10 minutes and 1 h for pERK and 1 h for EGR-1 expression with 100 µM carbachol or vehicle. Data are representative of at least three independent experiments. Scale bar: 50 µm.

We have previously shown that short-term mAChR activation in SK-N-SH cells resulted in the activation of ERK1/2 and the induction of EGR-1 [27]. Here we investigated whether the M1 mAChR signals similarly in HEK293 cells. HEK293-M1 treated with 100 µM of the muscarinic agonist carbachol mediated an increase in phosphorylation of ERK1/2 in all M1 expressing cells which is clearly evident at 10 mins after carbachol addition, and also highly elevated after 1 hr. Carbachol also increased induction of EGR-1 after 1 h (Fig. 2), which was evident in 100% of the cells. Basal levels of pERK and EGR-1 were low in vehicle treated HEK293-M1 expressing cells. Control HEK293-Vec cells stimulated with 100 µM carbachol exhibited a small signalling response, most likely due to the low level of endogenous M3 mAChR expression by HEK cells. This demonstrates that the M1 receptors are functional and signal as predicted.

### 3.3 Acute Activation of EGR-1 by M1 Receptors is ERK Dependent

**Figure 3:**
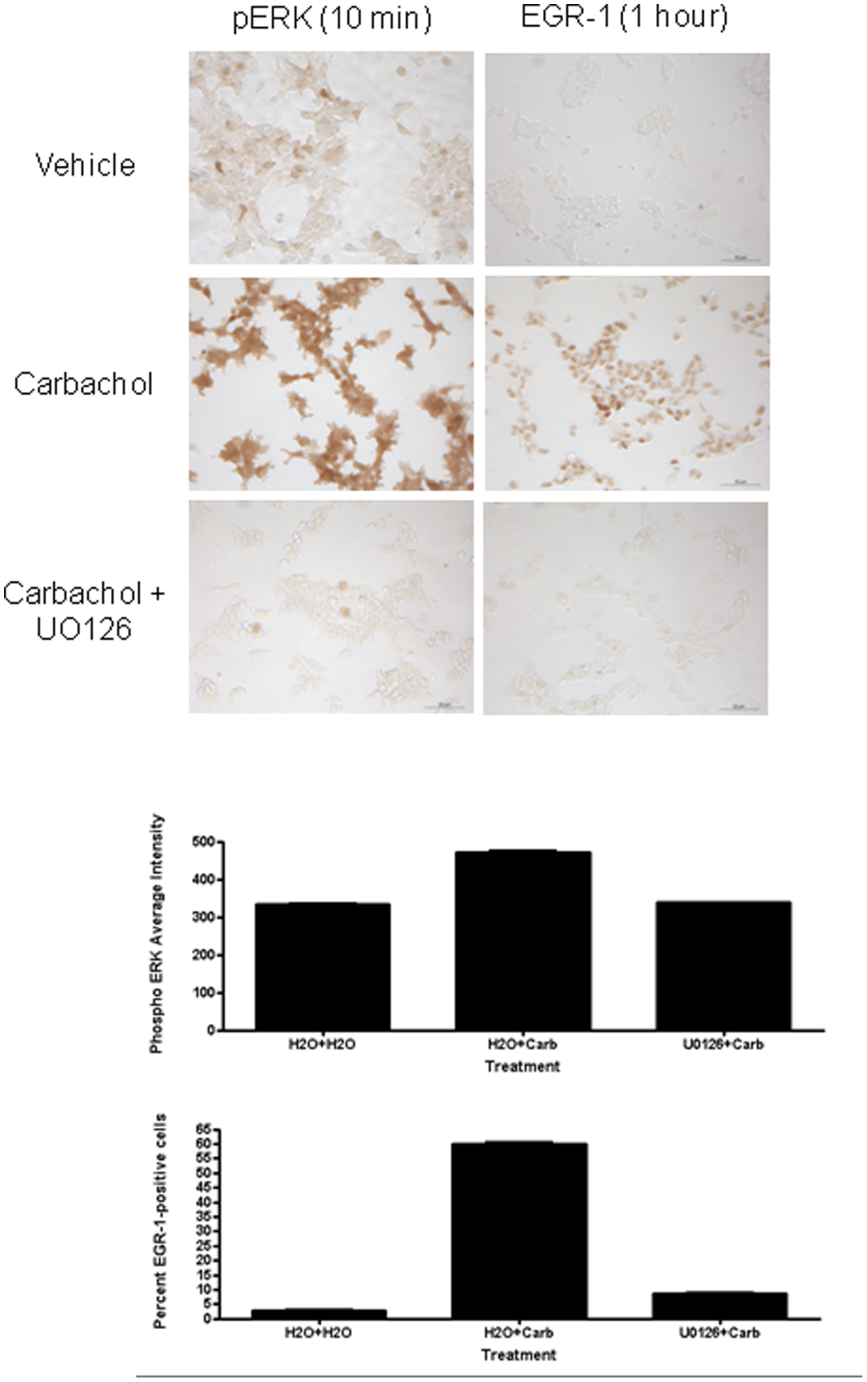
Acute activation of EGR-1 by M1 receptors is ERK dependent in HEK293-M1 cells. HEK293-M1 cells were pretreated with 5 µM UO126 (or vehicle) for 30 mins prior to the addition of 100 µM carbachol for the activation of pERK (10 mins) and EGR-1 (1 h). Data are representative of at least three independent experiments. Scale bar: 50 µM. Graphs show quantification of the induction and reversal of pERK and EGR-1 with U0126.

We have previously found in certain cell types that activation of specific transcription factors (e.g. EGR-1 induction) requires an intact MEK-ERK signalling pathway [27]. Here we used the MEK-selective inhibitor UO126 to prevent phosphorylation of ERK in HEK293-M1 cells. As shown in Figure 3, M1 stimulation by carbachol results in a rapid, strong activation of ERK, which is completely inhibited by UO126 treatment. In addition, ERK activation was required for M1 mediated acute induction of EGR-1 observed at 1 hr (Fig. 3).

### 3.4 Chronic M1 mAChRs Activation Induces HEK293-M1 Cell Death

**Figure 4:**
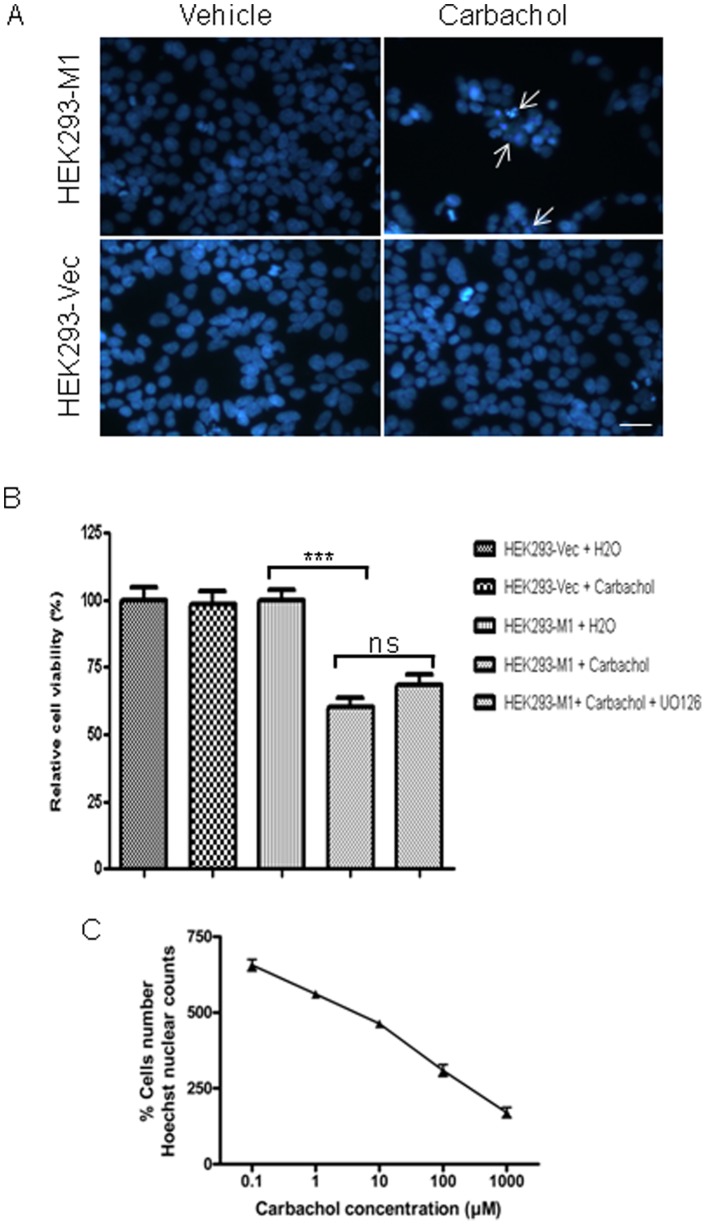
Chronic M1 mAChRs activation induces HEK293-M1 cell death. (A) Hoechst staining of HEK293-M1 and HEK293-Vec treated with water control (vehicle) or 100 µM carbachol for 24 h. The arrows point to condensed (possibly apoptotic) nuclei that were present in carbachol-treated HEK293-M1 cells. Note the substantial cell loss in the M1 expressing HEK cells, but not in the vector transfected HEK cells. Scale bar: 25 µm. (B) The extent of cell loss was investigated further using the MEK-inhibitor UO126. Hoechst stained cells were quantified using Discovery-1 and Metamorph analysis. Statistical significance: *** denotes p<0.001 compared with all other conditions. Note that UO126 did not significantly affect the carbachol response (ns) (C) HEK293-M1 cell counts across a range of 24 h carbachol treatments also showed that cell death occurred in a concentration-dependent manner. Data are representative of at least three independent experiments.

To investigate the effect of chronic M1 mAChR-activation on HEK293 cell viability, 100 µM carbachol was added to the cells for 24 h. Surprisingly, stimulation with carbachol resulted in a notable reduction in cell numbers but only in the M1 expressing HEK cells, not the HEK293-Vec control cells. Hoechst staining revealed the presence of apoptotic looking nuclei in HEK293-M1 cells 24 h after carbachol treatment (Fig. 4A). Discovery-1 and Metamorph analysis was used to quantify the number of cells following 24 h treatment with carbachol. This confirmed that carbachol-treated HEK293-M1 cells showed significantly reduced cell numbers (p<0.001, Fig. 4B) compared with those that were vehicle-treated. There was no effect of carbachol on cell number in the HEK293-Vec cells, demonstrating an M1 receptor dependent effect. Interestingly, UO126 only increased cell viability slightly, however, this small effect was not statistically significantly (Fig. 4b). The reduction in HEK293-M1 cells was concentration-dependent with increasing concentrations of carbachol mediating further decreases in HEK293-M1 cell numbers (Fig. 4C). Overall, this observation was surprising as it was initially predicted that M1 activation would be protective.

### 3.5 Analysis of Carbachol-mediated Cell Death using xCELLigence Technology

**Figure 5:**
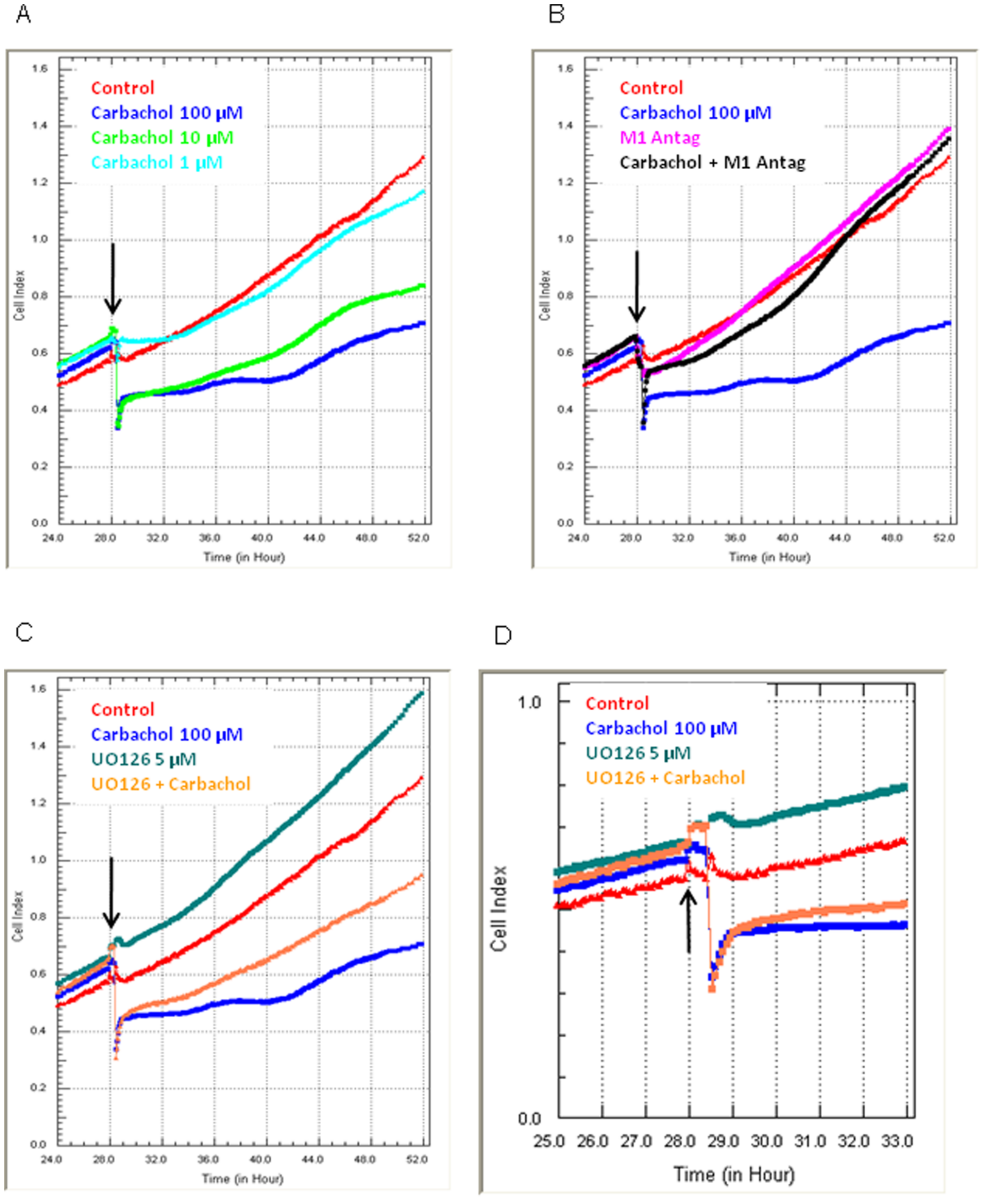
Analysis of carbachol mediated cell death using xCELLigence technology. HEK293-M1 cells were stimulated with various concentrations of carbachol (A), in the presence (or absence) of a M1 selective antagonist (B), and with 5 µM UO126 (C and D). Panel D shows the period immediately following drug addition to highlight the immediacy of the carbachol response. In each graph the black arrow shows the time point the respective drugs were added. Cellular measurements are made continuously in real time, where the level of total cell adhesion is represented as Cell Index. Higher Cell Index levels represent more adhesion, whereas a reduction in Cell Index represents a loss of adhesion. In all four graphs the red and blue Cell Index curves are the same and represent vehicle-treated and 100 µM carbachol-treated HEK293-M1 cells. The reduction in adhesion mediated by carbachol is immediate, concentration-dependent, blocked by M1 antagonist, but not blocked by UO126 (MEK blocker). xCELLigence data show representative Cell Index curves of at least six independent experiments.

Having established that M1 signalling resulted in cell death we next used xCELLigence biosensor technology to profile the changes in HEK293-M1 cells acutely and over the 24 hours following carbachol treatment. This was conducted to ascertain whether the cell compromise was immediate or occurred over a more gradual time course. Treatment of HEK293-M1 cells with carbachol resulted in a concentration dependent reduction in cell-adhesion, which was both immediate and sustained across the entire 24 hours following carbachol administration (Fig. 5A). This indicated that the effects of M1 activation on cell viability were more immediate than earlier thought. In addition, the xCELLigence Cell Index values did not reach 0.0, indicating that not all M1 expressing cells were lost following carbachol treatment, consistent with earlier observations (see Fig. 4). In addition, the profile of the Cell Index curve in the 10 µM and 100 µM carbachol treated cells shows the Cell Index measurements gradually increasing (∼44 hrs). This increase is consistent with the surviving cells continuing to proliferate with time. The Cell Index value 24 hours post carbachol treatment (100 µM) is ∼0.7, whereas in the control treated M1 cells the Cell Index measurement is ∼1.3; indicative of a 40–50% cell loss. The reduction in cellular adhesion (interpreted here as cell loss) mediated by 100 µM carbachol was completely blocked by pre-treatment with the M1 receptor antagonist, which had no appreciable effects on its own (Fig. 5B). The MEK inhibitor UO126 increased HEK293-M1 Cell Index values, suggesting that it improved adhesion and/or viability. This increase was also observed in the presence of 100 µM carbachol. In contrast to the M1 antagonist, UO126 failed to completely block the cell loss, again consistent with the observation from Discovery 1 analysis of cell viability (Fig. 5C, D).

### 3.6 Chronic M1 mAChRs Activation Induces Cleaved-caspase 3 in HEK293-M1 Cells

**Figure 6:**
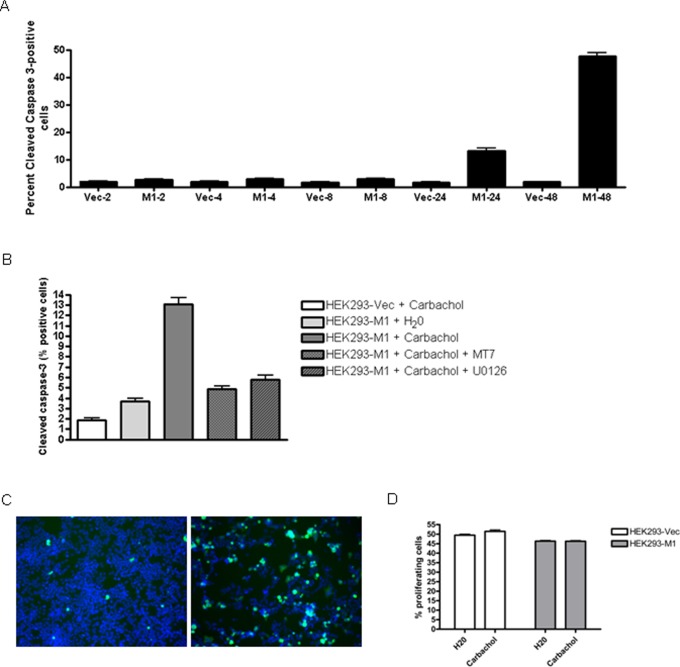
M1 mAChR activation induces cleaved-caspase 3 but does not induce changes in proliferation. HEK293-M1 and HEK293-Vec cells were stimulated across a 48 hour time course (2, 4, 8, 24, and 48 hours) with 100 µM carbachol (A). Induction of cleaved caspase-3 was evident following carbachol but only in the HEK293-M1 (M1) expressing cells. The induction was statistically significant at 24 hours and by 48 hours almost 50% of the cells expressed cleaved caspase 3. In contrast, there was no expression of cleaved caspase 3 in HEK-Vector (Vec) expressing cells. (B. To determine whether this expression was M1-mediated and MEK-mediated HEK293-M1 cells were incubated with the M1 antagonist MT7 or the MEK blocker U0126 and then challenged with carbachol. Induction of cleaved caspase 3 by carbachol was strongly inhibited by both drugs indicating that it was M1- and MEK-mediated. C. Photomicrographs of cleaved caspase 3 in HEK293-M1 cells 48 hours after vehicle (left image) or carbachol (right image) addition showing strong induction of cleaved caspase 3 in carbachol treated cells. (D) HEK293-Vec cells and HEK293-M1 cells were stimulated with 100 µM carbachol or vehicle for 24 hours, where BrdU was included during the final hour of treatment. BrdU incorporation revealed that the carbachol mediated reduction in cell numbers was not as a consequence of influencing proliferation. BrdU positive cells are as a percentage of the total cells counted to represent percentage proliferating cells. Data are representative of at least three independent experiments.

To better understand the mechanism of M1 mAChR mediated cell death, we investigated the expression of cleaved caspase 3 following carbachol treatment. Cleaved caspase 3 expression was induced in the carbachol treated HEK293-M1 cells (p<0.001), but was only present in less than 15% of M1 HEK cells at significant levels 24 hours after carbachol treatment. Forty-eight hours after carbachol treatment almost 50% of the cells expressed cleaved caspase 3 (Figure 6A, C). This response was not observed in HEK293-Vec cells (p<0.001). M1 receptor antagonist (MT-7) greatly reduced cleaved caspase 3 expression, as did MEK blockade with U0126 (Figure 6B). Cleaved caspase 3 is a consequence of the M1 receptor insult but only occurs in a minor population of cells (<15%) at 24 hours after carbachol addition which is much less than the almost 50% loss of cell number at this time-point (Figure 4B). Thus, the low expression of cleaved caspase 3 at 24 h cannot account for the rapid loss of cell number caused by carbachol at this time-point. However, by 48 hours almost 50% of the carbachol treated HEK-M1 cells expressed cleaved caspase 3 and this was also effectively reduced by both the M1 antagonist and by U0126 (data not shown). Therefore, the late expression of cleaved caspase 3 may contribute to longer term reductions in cell viability of these cells.

As mAChRs regulate proliferation in many cell types [28]–[31], we tested whether M1 mAChR activation in HEK293 cells altered proliferation. Twenty-three hours following carbachol treatment, 10 µM BrdU was added to the culture and then 1 h later the cells were fixed, and BrdU immunostaining was visualised. The percentages of BrdU-stained cells were quantified using Discovery-1 and Metamorph. We found that the percentage of cells nominally in S phase of the cell cycle did not change between treatments (Fig. 6D), further confirming that the reduction in carbachol-treated HEK293-M1 cells was through cell death rather than through a reduction in proliferation.

### 3.7 HEK293 Expressing CB1 Receptors are Not Killed by Chronic CB1 Activation

Because M1 mAChR activation had such a marked effect on cell viability (to our surprise) we investigated this further to determine whether the observation was common for other GPCRs. As HEK cells are widely used for acute signalling responses of numerous GPCRs, we used HEK cells transfected with the cannabinoid receptor 1 (CB1) [32] to see whether CB1 activation also led to apoptosis in HEK293 cells. HEK293-CB1 cells were stimulated with the synthetic cannabinoid agonist HU210, at a concentration of 100 nM for 24 h, but this did not lead to any cell death when compared to the vehicle control or a carbachol-treated control (data not shown).

### 3.8 Chronic M1 mAChR Activation Results in Prolonged Activation of pERK and EGR-1

**Figure 7:**
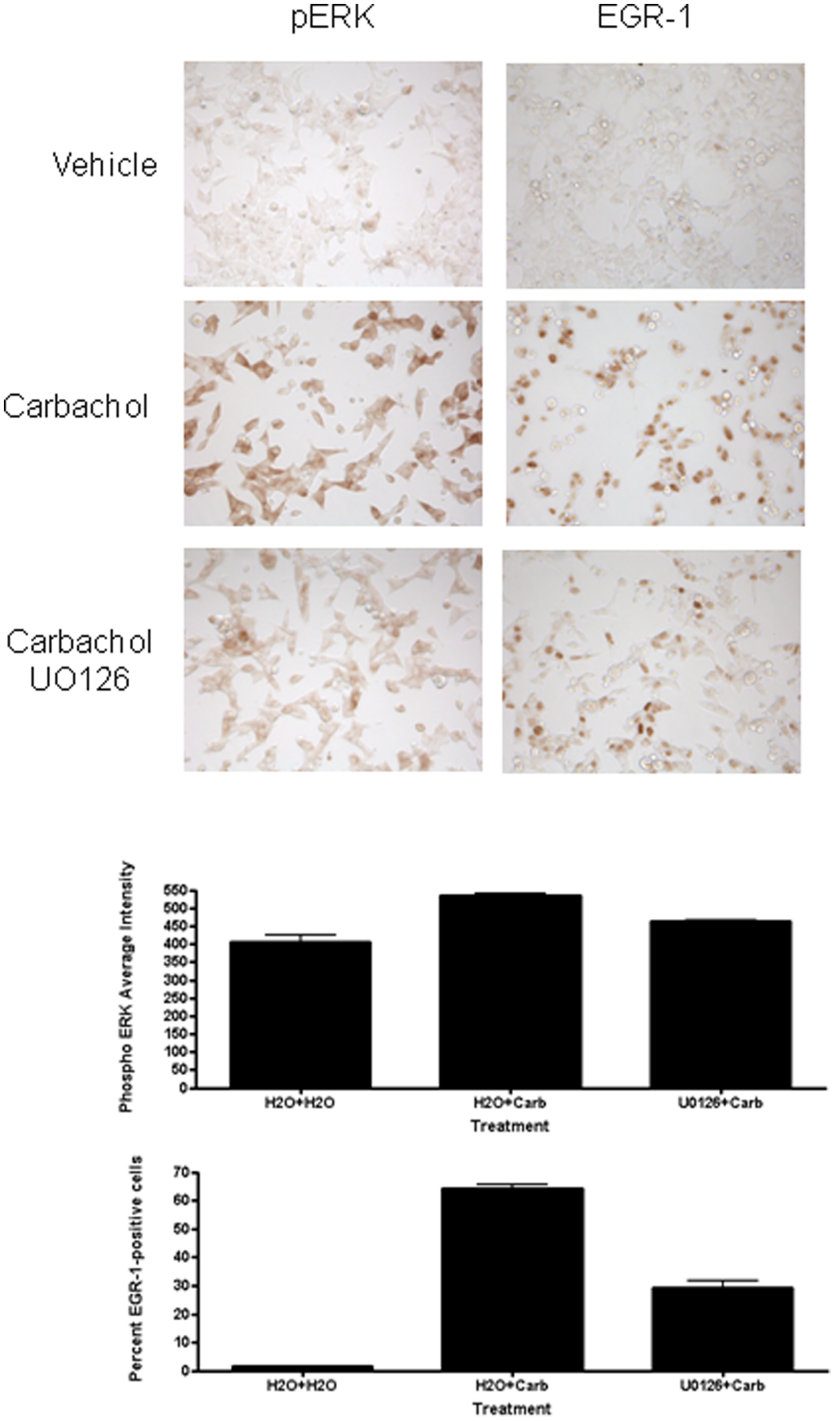
Activation of pERK and EGR-1 is still evident 24 hours following initial carbachol treatment. HEK293-M1 cells were stimulated for 24 h with vehicle or 100 µM carbachol as indicated. Then the cells were fixed and stained for pERK and EGR1. Note the high uniform activation of pERK and EGR-1. The lower panels show the effect of pretreatment (30 minutes) with 5 µM UO126, which shows that the aberrant signalling present at 24 h is independent of the MEK signalling pathway. Graphs show quantification of the induction and reversal of pERK and EGR-1 with U0126.

**Figure 8:**
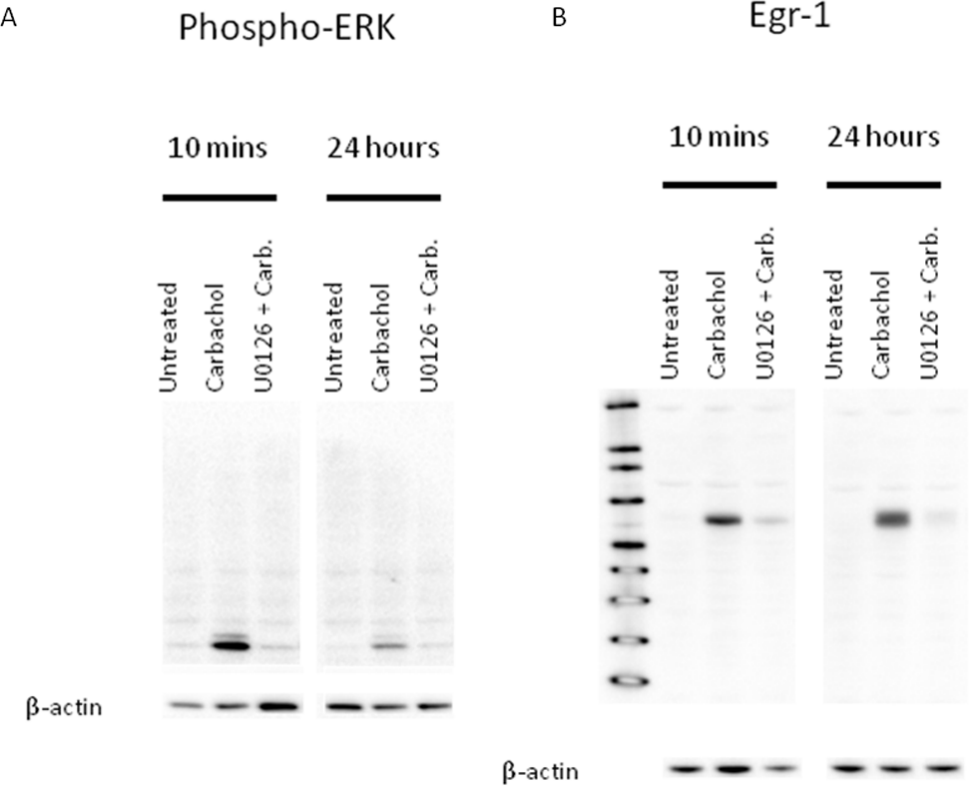
Western blots of pERK (A) and EGR-1 (B) at 10 min, 1 hr and 24 hours after Carbachol +/− U0126. A, pERK Western showing ERK phosphorylation at 10-min and 24 hours after addition of carbachol to HEK293-M1 cells. The MEK blocker U0126 completely inhibited this ERK phosphorylation. B, EGR-1 induction at 1 hour and 24 hours after addition of carbachol to HEK293-M1 cells. The MEK blocker U0126 completely inhibited this EGR-1 induction.

In our previous work we found that the carbachol-mediated induction of EGR-1 in SK-N-SH neuroblastoma cells was transient, tapering off after 1 h, returning to basal levels within 8 h [27]. However, in the present study we discovered that phosphorylation of ERK and the induction of EGR-1 were prolonged in HEK293-M1 cells, with high levels still present in the surviving cells at 24 h (Fig. 7). The level of EGR-1 induced acutely by M1 activation by carbachol was dependent upon pERK. Similarly, the induction of EGR-1 at 24 h after carbachol administration was also reduced, although not completely inhibited, by UO126. To confirm these immunocytochemical results we also performed Western blots for pERK and EGR-1 at 10 min, 1 and 24 hours after carbachol addition to HEK293-M1 cells treated with carbachol, as well as U0126 (Fig. 8A, B) and these western results corroborated our immunocytochemical analyses.

## Discussion

HEK293 cells have been used extensively to generate stable cell lines to study G protein-coupled receptors, such as muscarinic acetylcholine receptors (mAChRs) and cannabinoid receptors [32]–[37]. These cells are readily transfected and provide powerful cellular tools for studying receptor biology and drug screening. Our studies have shown that stable cells lines expressing human M1 mAChRs show many of the characteristics of cells expressing the endogenous receptors – receptor internalisation with agonist exposure and phosphorylation of ERK and induction of EGR-1 after transient exposure to the agonist carbachol. Furthermore, the rapid carbachol-mediated induction of EGR-1 was blocked by the MEK blocker U0126, which is consistent with our previous studies [27]. However, when carbachol is added to these cells for prolonged periods (24 h) the cells show aberrant signalling with sustained phosphorylation of ERK and induction of EGR-1 maintained at high levels for prolonged periods of time. This prolonged activation of EGR-1 was also strongly inhibited by the MEK blocker U0126. In contrast, vector-transfected HEK293 cells (which endogenously express M3 receptors) show only the transient activation of ERK and EGR-1, consistent with previous reports [27], [38].

In contrast to these results with M1 mAChRs we have previously found that Neuro2a cells endogenously expressing the cannabinoid CB1 receptor show only a transient activation of ERK and EGR-1 after agonist (HU210) exposure and found no evidence of CB1-mediated cell death [39]. Also CB1 receptor activation in transfected HEK cells does not result in cell death either following acute or chronic stimulation [32]. The reasons for this aberrant M1 signalling are currently not clear. Our stable cell lines were fully characterised using specific antibodies to the M1 receptor and HA-tag, which showed that we had good cell surface expression which internalised properly following agonist exposure. This characterisation, along with the pharmacological data indicated that the receptors were indeed M1.

**Figure 9:**
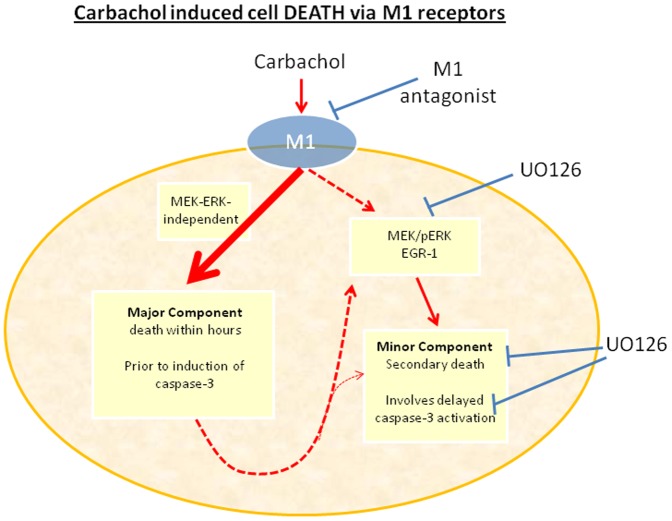
Schematic diagram showing that M1-induced cell death involved two components. The major component of death was ERK-independent and occurred within hours of carbachol activation of the cells. This pathway was not blocked by the MEK inhibitor UO126 and occurred prior to activation of cleaved caspase-3. In contrast the minor component, which was slower and more progressive, was blocked by UO126 and associated with cleaved caspase-3 expression. Both pathways were fully blocked by the M1 antagonist and only occurred in HEK-M1 cells stably expressing M1 receptors.

In addition, to the aberrant signal transduction cascades, we noted a significant loss of cells during the initial 24 hour exposure to carbachol. This was initially observed during standard cell counting assays and investigated further with time-lapse microscopy (data not shown) and xCELLigence technology to reveal the true time course of carbachol effects on cell viability. xCELLigence technology directly measures cell adhesion and is an ideal platform to investigate acute and chronic cellular events, especially those associated with cell death [40]. In this study, xCELLigence reveals that the effect of carbachol occurs within the first couple of hours and the loss of approximately 50% of the total adhesion is consistent with the cell viability and cell count data at 24 hours. This effect was not seen in vector-expressing HEK293 cells, HEK293 cells stably expressing the CB1 receptor or human neuroblastoma cells. Furthermore, the apoptosis was M1 receptor mediated, as the M1 antagonist blocked the death of HEK293-M1 cells. However, the rapid cell death was not inhibited by U0126 and therefore was not MEK-ERK mediated. Thus, the mechanism of the rapid cell death caused by carbachol is not clear but also does not appear to be caspase 3 mediated because the cleaved caspase 3 expression occurred too late (between 8–24 hrs) to account for the initial rapid cell death (occurring within a few hours). However, cleaved caspase 3 may account for cell death at later (>24 h) time-points, and this might be mediated by MEK because the MEK blocker effectively inhibited this late cleaved caspase 3 expression. Thus, the cytotoxic effects of carbachol on HEK293-M1 cells are complex and require further study to understand their mechanisms (see Figure 9).

To the best of our knowledge, this is the first study reporting M1 mAChR-mediated cell death in HEK293 cells. There have been numerous studies using HEK293 as a cell model to study mAChR, but most use short-term agonist treatments (including carbachol) mainly to investigate cell signalling pathways. Stope *et al.* however, treated HEK293-M3 cells with carbachol for 24 h, but did not report any occurrences of apoptosis [35]. In rat primary retinal cultures, the M1 mAChR provided effective protection against glutamate-induced neuronal apoptosis through several mechanisms [8]. It was also shown that retinal ganglion cells had increased survival in culture when the M1 mAChR was activated [10]. In transfected PC12 cells, the M1 mAChR rescues the cells from serum starvation by inhibiting caspase activity [9]. Recently, however, Reina et al [41] demonstrated that activation of M1 and M3 muscarinic receptors with the agonist pilocarpine lead to apoptosis of human fibroblast cells derived from skin. This result suggests that activation of endogenous M1 receptor is capable of inducing apoptosis. Furthermore, other studies of the related M3 muscarinic receptor have shown both anti-apoptotic and also pro-apoptotic actions [3], [4], [7] [42], [43] [5], [6], [44], [45]. The reasons for these different results are presently unclear, but it is likely that the M3 mAChR has dual roles in controlling both cell death and survival, which is independent of the cell line, and these cell fate decisions are possibly controlled through the surrounding conditions in which the M3 mAChR is expressed in a manner more complex than first thought. Whether similar processes are involved in the effects of the M1 receptor, as demonstrated by our data, is not known.

In conclusion, this study shows that M1 mAChR activation causes aberrant signalling events and cell death in HEK293 cells. Given that the HEK293 cell line is widely used as a pharmacological tool for studying G-protein coupled receptors, including mAChRs, these results indicate that caution must be heeded using this model. Although we have shown that acute activation of these HEK-M1 receptors causes signalling and internalization changes consistent with previous studies the aberrant chronic signalling suggests that the receptor is not acting in a physiological fashion. Thus, compounds developed based on this transfected system may not act as expected on physiological M1 receptors. The most obvious reason for these results is that the carbachol mediated death is occurring in cells with supra-physiological levels of M1 receptors. This is a known aspect of HEK cells and other heterologous expression systems for the study of GPCR. Our studies highlight the importance of investigating the longer term events occurring in these types of models and advocate the usefulness of xCELLigence technology in these studies. Identifying how and why activation of the M1 mAChR mediate aberrant signalling and apoptosis in these cells may lead to a better understanding of how mAChRs regulate cell fate decisions. In particular, it might be possible to develop agonists and antagonists that mimic or block pro-apoptotic or anti-apoptotic pathways following M1 receptor activation.
